# Supplementation of Specific Collagen Peptides Following High-Load Resistance Exercise Upregulates Gene Expression in Pathways Involved in Skeletal Muscle Signal Transduction

**DOI:** 10.3389/fphys.2022.838004

**Published:** 2022-04-05

**Authors:** Christoph Centner, Simon Jerger, Alistair Mallard, Anna Herrmann, Eugenia Varfolomeeva, Sandra Gollhofer, Steffen Oesser, Carsten Sticht, Norbert Gretz, Per Aagaard, Jakob L. Nielsen, Ulrik Frandsen, Charlotte Suetta, Albert Gollhofer, Daniel König

**Affiliations:** ^1^ Department of Sport and Sport Science, University of Freiburg, Freiburg, Germany; ^2^ Praxisklinik Rennbahn, Muttenz, Switzerland; ^3^ Australasian Kidney Trials Network, Centre for Health Services Research, Faculty of Medicine, University of Queensland, Brisbane, QLD, Australia; ^4^ CRI, Collagen Research Institute, Kiel, Germany; ^5^ Medical Faculty Mannheim, University of Heidelberg, Heidelberg, Germany; ^6^ Department of Sports Science and Clinical Biomechanics, Research Unit for Muscle Physiology and Biomechanics, University of Southern Denmark, Odense, Denmark; ^7^ Geriatric Research Unit, Department of Geriatric and Palliative Medicine, Copenhagen University Hospital Bispebjerg and Frederiksberg, Copenhagen, Denmark; ^8^ Geriatric Research Unit, Department of Medicine, Copenhagen University Hospital Herlev and Gentofte, Copenhagen, Denmark; ^9^ Department of Nutritional Science, Institute for Nutrition, Exercise and Health, University of Vienna, Vienna, Austria; ^10^ Centre for Sports Science and University Sports, Institute for Nutrition, Exercise and Health, University of Vienna, Vienna, Austria

**Keywords:** gene expression, pathway analysis, collagen peptides, resistance exercise, KEGG enrichment analysis

## Abstract

Previous evidence suggests that resistance training in combination with specific collagen peptides (CP) improves adaptive responses of the muscular apparatus. Although beneficial effects have been repeatedly demonstrated, the underlying mechanisms are not well understood. Therefore, the primary objective of the present randomized trial was to elucidate differences in gene expression pathways related to skeletal muscle signal transduction following acute high-load resistance exercise with and without CP intake. Recreationally active male participants were equally randomized to high-load leg extension exercise in combination with 15 g CP or placebo (PLA) supplementation. Muscle biopsies from the vastus lateralis muscle were obtained at baseline as well as 1, 4 and 24 h post exercise to investigate gene expression using next generation sequencing analysis. Several important anabolic pathways including PI3K-Akt and MAPK pathways were significantly upregulated at 1 and 4 h post-exercise. Significant between-group differences for both pathways were identified at the 4 h time point demonstrating a more pronounced effect after CP intake. Gene expression related to the mTOR pathway demonstrated a higher visual increase in the CP group compared to PLA by trend, but failed to achieve statistically significant group differences. The current findings revealed a significantly higher upregulation of key anabolic pathways (PI3K-Akt, MAPK) in human skeletal muscle 4 h following an acute resistance training combined with intake of 15 g of specific collagen peptides compared to placebo. Further investigations should examine potential relationships between upregulated gene expression and changes in myofibrillar protein synthesis as well as potential long-term effects on anabolic pathways on the protein level.

## Introduction

Human skeletal muscle mass accounts for up to 35% of total body weight (Janssen et al., 2000) and serves vital functions such as generating force and thus enabling locomotion. Additionally, skeletal muscle metabolism comprises numerous metabolic processes including energy storage (Hultman et al., 1971), energy consumption and endocrine functioning (Hoffmann and Weigert, 2017). Thus, the preservation or even increase of skeletal muscle mass and strength are of fundamental importance to sustain and improve many physiological functions including a healthy aging process with the longest possible maintenance of activities of daily living (Wang et al., 2020). Resistance exercise in combination with protein ingestion is known to be a potent stimulator of skeletal muscle protein synthesis and the timely coordinated combination of both has been demonstrated to augment net muscle protein synthesis (Burd et al., 2009; Wirth et al., 2020).

Previous research indicated that the beneficial effects of protein supplementation (in particular whey protein (Moore et al., 2011; Areta et al., 2013; Morton et al., 2018; Hamarsland et al., 2019)) on muscle protein synthesis are predominantly mediated by the essential amino acid leucine, which augments intramuscular muscle protein synthesis *via* the mechanistic target of rapamycin (mTOR) complex and its downstream regulators (Drummond and Rasmussen, 2008; Moberg et al., 2016). Additionally, *via* exercise-induced growth factor secretion, the stimulation of the PI3k-Akt pathway has repeatedly been shown to induce protein synthesis *via* activation of its downstream targets (Bodine et al., 2001). Independently of mTOR activity, resistance exercise can also activate the mitogen-activated protein kinase (MAPK) pathway. Previous researchers (Moore et al., 2011) speculated that MAPK might facilitate the stimulation of myofibrillar protein synthesis by regulating proteins which mediate the initiation and elongation stages of mRNA translation (Wang et al., 2001; Roux et al., 2007).

Based on clinical and preclinical studies on the acute and longitudinal muscular responses to resistance exercise and protein consumption, whey protein (Moore et al., 2011; Areta et al., 2013; Hamarsland et al., 2019), soy (Darren et al., 2006; Tang et al., 2009), casein (Tipton et al., 2004; Tang et al., 2009) or collagen peptides (Jendricke et al., 2019; Kirmse et al., 2019; Zdzieblik et al., 2021) have been receiving increased attention over the past 2 decades.

Collagen peptides (CP) in combination with resistance training have previously been shown to improve muscle function and increase fat-free mass beyond the levels of resistance training alone (Zdzieblik et al., 2015; Jendricke et al., 2019; Kirmse et al., 2019), although some studies found less pronounced effects on muscle protein synthesis (Oikawa et al., 2020) and muscle mass compared to whey protein (Jacinto et al., 2022). In fact, however, Jacinto et al. (2022) did not find significant group differences with regard to performance parameters, which is practically relevant for the effectiveness of training. To date, little is known about the underlying mechanisms on a cellular level responsible for these observations. While CPs are rich in amino acids such as proline, glycine and arginine (Zdzieblik et al., 2015), the amount of e.g., leucine is low, which might lead to the conclusion about limited anabolic potential. Yet, *in vitro* data by Kitakaze et al. (2016) have indicated that the collagen derived dipeptide hydroxyprolyl-glycine promotes activation of the PI3K-Akt-mTOR signaling pathway. In a recent clinical investigation, Oertzen-Hagemann and others (2019) analyzed the skeletal muscle proteome before and after 12 weeks of resistance training in combination with CP supplementation. The authors demonstrated that anabolic signaling pathways were upregulated with CP combined with resistance training, as indicated by a higher upregulation of the MAPK pathway in response to CP supplementation compared to placebo intake (Oertzen-Hagemann et al., 2019).

Based on these findings (Kitakaze et al., 2016; Oertzen-Hagemann et al., 2019), the present study aimed to investigate the acute effects of CP in combination with resistance exercise on gene expression in pathways known to be activated in muscular remodeling, such as MAPK, mTOR and PI3K-Akt. Specifically, this study examined acute changes in gene expression in relation to a single high-load resistance exercise. Investigating these short-term responses is crucial for understanding basic physiological mechanisms of CP action in combination with resistance exercise.

## Materials and Methods

### Participants

In total, *n* = 30 male participants were included in this randomized-controlled double-blinded study. To decrease variability in anabolic responses, strict inclusion and exclusion criteria were utilized. Participant age was limited to between 18 and 29 years. All participants were currently recreationally active (∼150 min/wk of moderate or 75 min/wk of vigorous exercise (e.g., team sports, cycling)). Additionally, participants’ BMI was within 18.5 kg/m^2^ and 25 kg/m^2^. Participants diagnosed with any acute or chronic diseases, cardiac conditions, or metabolic disorders were excluded from the study.

The local ethics committee approved all procedures of this study, which all adhered with the Declaration of Helsinki. All participants were informed about the study procedures and any potential risks involved in the study and provided written informed consent (DRKS00027112).

### Study Design

A randomized, controlled, double-blinded design was implemented to investigate the effects of CP ingestion combined with acute resistance exercise on skeletal muscle anabolic gene expression.

One week before the acute exercise trial, participants visited the laboratory and underwent a preliminary screening, which included a survey of the complete medical history and a general medical examination to ensure compliance with the study-specific inclusion criteria. After the assessment of eligibility, participants were randomly allocated into two groups either supplemented with CP or placebo. A random number generator was used for allocation sequence generation. All outcome assessors, statistical analysis group, as well as trainers and participants were blinded to group assignments.

### One-Repetition Maximum Assessment

One week prior to the exercise trial participants visited the laboratory and completed a familiarization session with the knee extensor training device (FREI AG, Kirchzarten, Germany). Additionally, a one-repetition maximum (1RM) assessment of the right knee extensors was completed. Before conducting the 1RM test, a warm-up consisting of two sets of ten repetitions at submaximal load followed by two additional sets of three to five repetitions were completed (Baechle and Earle, 2000). During all warm-up and testing procedures, repetitions were performed with a full range of motion from 90° knee flexion to full extension (180°). After warm-up, participants selected a weight close to their perceived maximum and performed one repetition. Weight was increased by 5–10% until the participants were not able to lift the weight through the full range of motion (Baechle and Earle, 2000; Patterson and Ferguson, 2011). Each attempt was followed by a rest period of 4 min to assure recovery and the final 1RM was achieved within five attempts.

### Experimental Protocol

**FIGURE 1:**
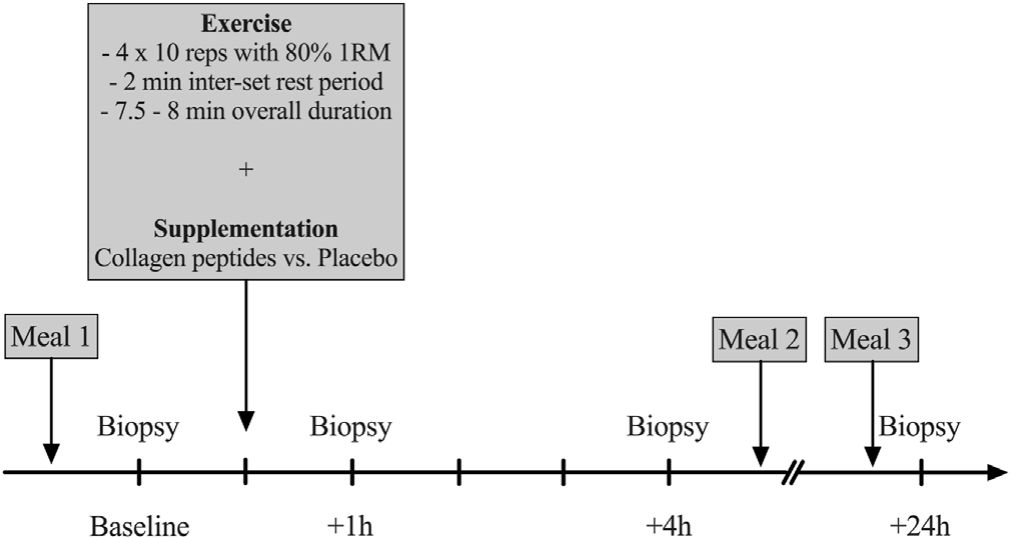
Overview on the experimental design (1RM = One repetition maximum, reps = repetitions).

After an overnight fast of 10–12 h, participants arrived at the laboratory between 7 and 8 am. To eliminate any bias of prior physical activity, all participants were instructed to refrain from any exercise 48 h prior to the trial and avoid certain means of transportation on their way to the laboratory (e.g., bicycle commute). After a resting period of 30 min, a muscle biopsy was obtained from the m. vastus lateralis (VL) muscle of the right leg (more details provided below). 60 min following this biopsy a high-load (80% 1RM) resistance exercise bout was completed (details provided below). Immediately after exercise participants consumed a beverage containing 250 ml of water and either 15 g of CP (Bodybalance^®^, Gelita AG, Eberbach, Germany) or PLA (silicon dioxide). CP and PLA products were similar in taste and appearance. Details on the specific CP amino acid composition are reported in (Table 1). Following the temporal dynamics of the uptake of collagen peptides (∼1–2 h after ingestion) (Shigemura et al., 2014), three additional biopsies were taken 1, 4 and 24 h after completion of the exercise bout (Figure 1). These time points have been frequently used in this context to assess intramuscular signaling cascades following exercise and nutritional interventions (Areta et al., 2013; Camera et al., 2015; Jackman et al., 2017). Between the first three biopsies, participants stayed at the laboratory and were instructed to rest.

**TABLE 1:** Amino Acid Composition of the CP supplement.

Amino acid	Weight (%)	Mol (%)
Hydroxyproline	11·3	9·6
Aspartic acid	5·8	4·8
Serine	3·2	3·4
Glutamic acid	10·1	7·5
Glycine	22·1	32·3
Histidine	1·2	0·8
Arginine	7·8	5·0
Threonine	1·8	1·7
Alanine	8·5	10·5
Proline	12·3	11·8
Tyrosine	0·9	0·5
Hydroxylysine	1·7	1·2
Valine	2·4	2·3
Methionine	0·9	0·9
Lysine	3·8	2·9
Isoleucine	1·3	1·1
Leucine	2·7	2·3
Phenylalanine	2·1	1·4

### Exercise Protocol

The exercise session was designed in accordance with protocols reported in previous studies investigating the acute effect of protein supplementation on muscle protein synthesis (Burd et al., 2010; Areta et al., 2013). All exercise was conducted using the FREI AG (Kirchzarten, Germany) knee extension machine. After a warm-up of two sets of five repetitions with 50 and 60% 1RM (Areta et al., 2013), four sets of 10 repetitions at 80% 1RM were completed with the right leg. Each set was separated by a 2-min resting period (Burd et al., 2010). The range of motion reached from 90° knee flexion to full extension (180°). The duration of the exercise session was ∼7.5 min.

### Diet

Individual dietary intake was recorded by all participants for 72 h before the experimental trial as previously described (Burd et al., 2010). Further, all participants were instructed to abstain from alcohol and caffeine for 48 h before the experiment (Camera et al., 2015). The subjects were instructed to maintain their habitual dietary intake until the evening prior to the experimental trial. At this point in time, participants were provided with pre-packed meals, i.e., dinner on the night before the first biopsy as well as lunch and dinner between the 4 and 24 h biopsy. The meals were designed to meet protein guidelines of 0.4 g per kg body weight per meal for resistance-trained athletes (Schoenfeld and Aragon, 2018) corresponding to 1.2–1.4 g per kg bodyweight per day (Tang et al., 2009; Konig et al., 2020), and complying with caloric requirements estimated using the Benedict Harris equation (PAL 1,6) (Moore et al., 2009). Consequently, baseline biopsies as well as 24 h biopsies were obtained following an overnight fasting (Hulmi et al., 2009).

### Muscle Biopsy Sampling

Muscle biopsies were obtained from the VL of the right leg using a 5-mm Bergström needle with manual suction following local anesthesia (2% Xylocitin) (Nielsen et al., 2012; Suetta et al., 2013). The location of all incision sites was randomized, and all incisions were 2–3 cm apart arranged in a parallelogram shape at the mid-portion of the VL muscle. After collection of the muscle tissue, the material was cleaned if there was excess blood using blotting paper and frozen in liquid nitrogen. Subsequently, all samples were stored at −80°C for later analysis (Nielsen et al., 2012; Suetta et al., 2013).

### RNA Sequencing

RNA was prepared using Trizol (Gibco) followed by additional purification using the RNeasy Mini Kit (Qiagen). RNA quality was checked with the Agilent 2,100 Bioanalyzer and the RNA 6000 Nano Kit (Agilent, Waldbronn). Samples with RNA integrity number (RIN) above 9.5 were used for RNA sequencing. The sequencing work was performed by BGI Tech Solutions Co. (Hong Kong, China). RNA sequencing was performed with BGISEQ-500, a new desktop sequencer developed by BGI. Using DNA nanoball and combinational probe anchor synthesis developed from Complete Genomics™ sequencing technologies, it generates short reads at a large scale. The RNA sequencing was performed with 50 M reads paired end. The raw and normalized data are deposited in the Gene Expression Omnibus database (http://www.ncbi.nlm.nih.gov/geo/; accession No. GSE195585).

### Statistical Analysis

Most of the statistical evaluations of the pre-to-post exercise changes in mRNA expression were completed with R and bioconductor using the NGS analysis plackage systempipe R (Backman and Girke, 2016). Quality control of raw sequencing reads was accomplished using FastQC (Babraham Bioinformatics). Low-quality reads were removed with trim_galore (version 0.6.4). The resulting reads were aligned to human genome version GRCh38. p13 from GeneCode and counted using kallisto version 0.46.1 (Bray et al., 2016). The count data was transformed to log2-counts per million (logCPM) using the voom-function from the limma package (Ritchie et al., 2015). The comparison of changes between CP and placebo was performed with the linear regression method using the limma package in R for every timepoint (1, 4, 24 h). A false positive rate of *α* = 0.05 with FDR correction was taken as the level of significance. Colored KEGG plots were made with the pathview package in R (Luo and Brouwer, 2013).

The pathway analysis was made with fgsea package (Sergushichev, 2016) and the enrichment browser package (Geistlinger et al., 2016) in R using the pathway information from KEGG database (https://www.genome.jp/kegg/pathway.html). The GSEA (gene set enrichment analysis) is a method to identify enriched pathways with up- or downregulated genes. Within this approach, enrichment scores (ES) are calculated, which reflect the degree to which a pathway is enriched with upregulated (positive ES) or downregulated (negative ES) genes (https://www.gsea-msigdb.org/gsea). This corresponds to up- or downregulated pathways. Normalizing the ES [normalized ES (NES)] by the mean of gene ES of all dataset permutations allows comparisons across gene sets. The gene pathway approach is of superior interpretational value compared to single gene expression analysis, since only the upregulation of a whole pathway (and thus the concert of upregulated single genes) facilitates specific functional consequences. As described above, the pathway composition is based on the KEGG database.

## Results

All participants successfully completed all aspects of the study (*n* = 15 CP, *n* = 15 PLA). There were no differences between groups for any anthropometric or exercise related measurements (Table 2).

**TABLE 2:** Baseline anthropometric characteristics (CP/PLA *n* = 15/15).

Variable	Group	(Mean ± SD)
Age (years)	CP	25.4 ± 2.4
	PLA	24.0 ± 2.6
Height (cm)	CP	177.9 ± 7.9
	PLA	178.5 ± 7.3
Weight (kg)	CP	73.3 ± 10.0
	PLA	71.2 ± 7.8
BMI (kg/m^2^)	CP	23.2 ± 2.3
	PLA	22.4 ± 2.4
Knee extensor, 1RM (kg)	CP	68.0 ± 15.8
	PLA	63.5 ± 6.1

### Gene Expression Analysis

**FIGURE 2:**
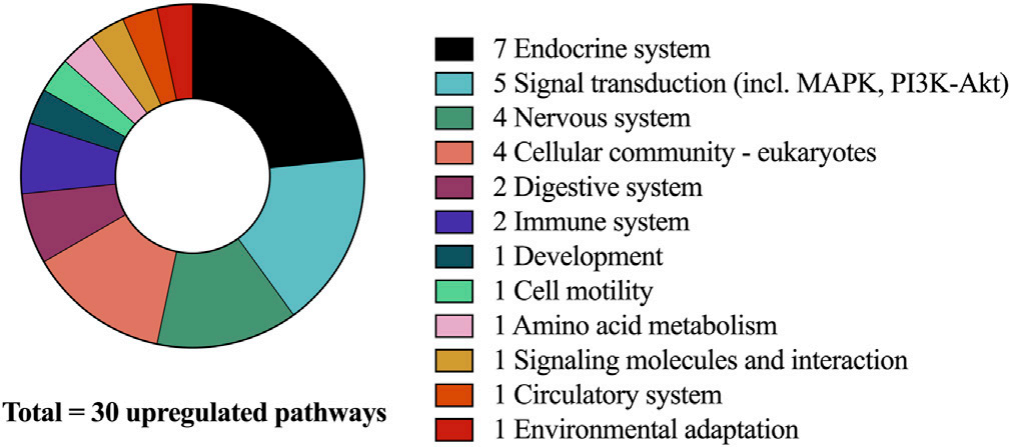
Upregulated pathways after CP supplementation compared to PLA at 4-h and associated sub-categories.

Across groups, the signaling pathways that were most upregulated demonstrated peak changes in mRNA expression at the 4 h timepoint. This is in accordance with previous studies investigating the effects of acute resistance exercise on gene expression (Oertzen-Hagemann et al., 2019). Overall, at this timepoint a total of 30 signaling pathways were found to be significantly upregulated (Figure 2).

### PI3K-Akt

**FIGURE 3:**
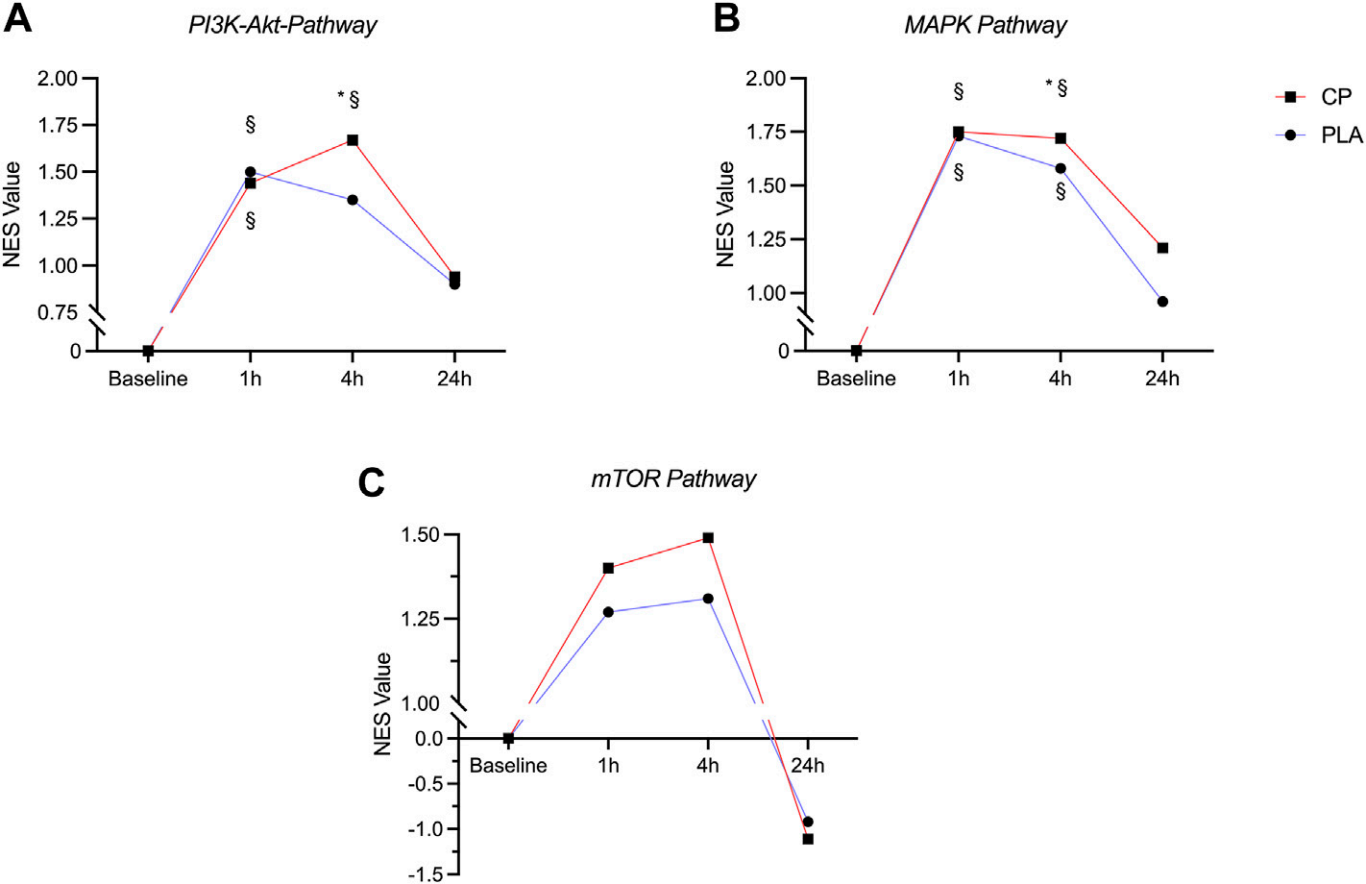
Time course of the normalized enrichment scores (NES) in the PI3K-Akt-Pathway **(A)**, MAPK-Pathway **(B)** and mTOR pathway **(C)** in the collagen peptide (CP, *n* = 15) and placebo (PLA, *n* = 15) group. § = significantly different from baseline (p^FDR^ < 0.05), * = significantly different from PLA (p^FDR^ < 0.05).

Compared to baseline, the PI3K-Akt-Pathway was significantly enriched with upregulated genes at 1 h for both CP and PLA (p^FDR^ < 0.05). At the 4 h timepoint, significant differences compared to baseline were observed for CP only (p^FDR^ < 0.05). These differences were significantly larger compared to PLA with normalized enrichment scores of 1.67 for CP and 1.35 for PLA. No significant differences between CP and PLA were observed at the 24 h timepoint (Figure 3A).

### MAPK

A significant upregulation of gene expression in the MAPK pathway was observed at 1 and 4 h post-exercise for both CP and PLA compared to baseline (p^FDR^ < 0.05). Significant group differences were observed at 4 h in favor of CP (p^FDR^ < 0.05). No significant upregulation of the MAPK gene expression pathway was seen after 24 h (Figure 3B).

### mTOR

Compared to baseline, the mTOR pathway was descriptively enriched with upregulated genes at 1 and 4 h post exercise for both CP and PLA, however after correction for multiple testing these changes did not reach statistical significance for neither CP nor PLA (p^FDR^ = 0.347) (Figure 3C).

## Discussion

The main purpose of the present study was to systematically investigate changes in gene expression following an acute bout of high-load resistance exercise combined with specific collagen peptides compared to placebo. This acute study design was chosen, since it best reflects the temporal dynamics of gene expression. The main findings indicated a significantly upregulated gene expression in key myocellular pathways (PI3K-Akt, MAPK) involved in protein synthesis and muscle hypertrophy following the ingestion of collagen peptides compared to placebo.

The PI3K-Akt pathway can be activated by several precursors including IGF-1 and regulates multiple intracellular processes including muscle hypertrophy (Glass, 2010) and collagen synthesis (Yokoyama et al., 2012). As a serine-threonine protein kinase, Akt is capable of inducing myofibrillar protein synthesis and inhibit the upregulation of muscle atrophy markers such as MuRF-1 or Atrogin-1 *via* phosphorylation of FOXO (Glass, 2010). In addition to the inhibition of atrophy-related markers, PI3K-Akt has been demonstrated to block myostatin and thus stimulate differentiation and skeletal protein synthesis *via* distinct physiological mechanisms.

**FIGURE 4:**
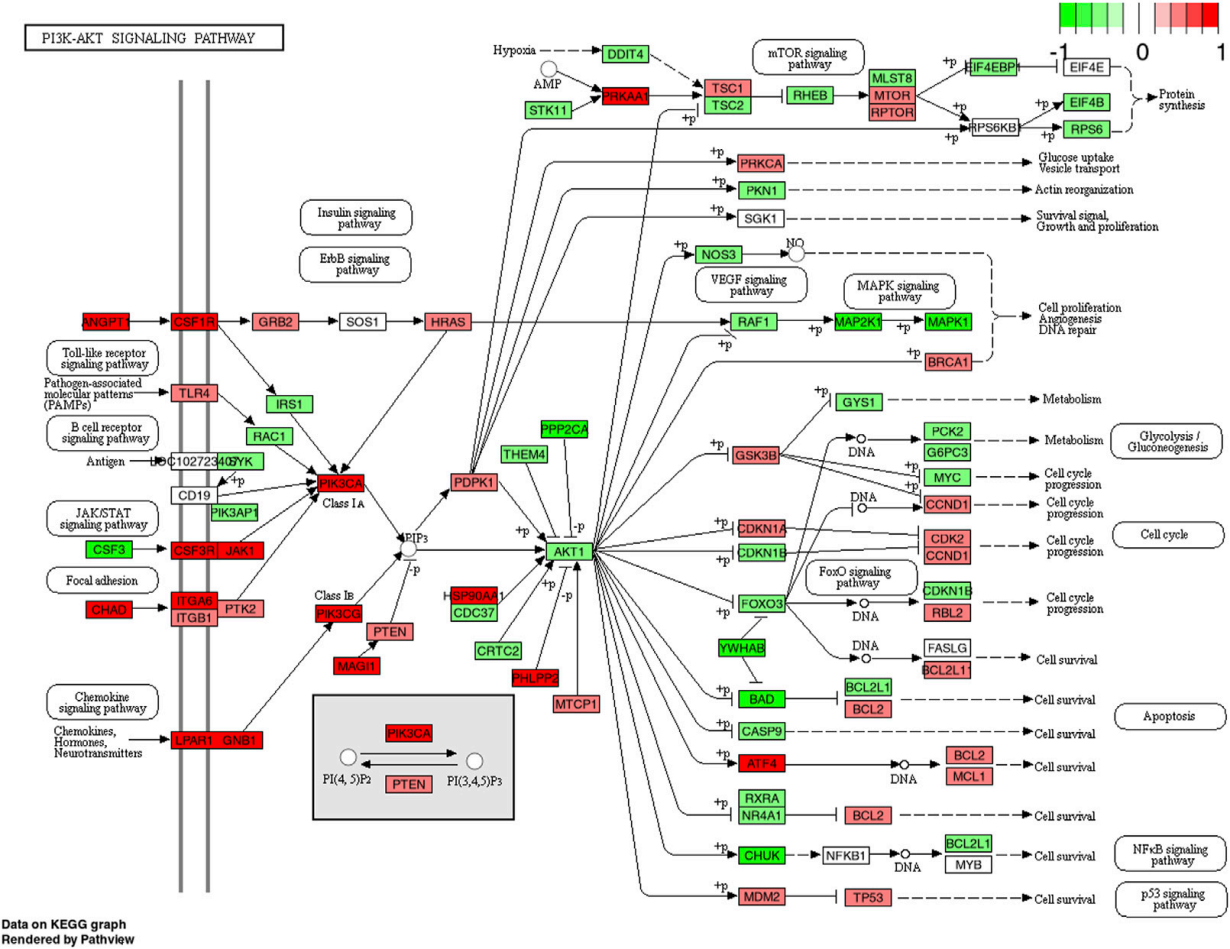
KEGG pathway of the PI3K-Akt signaling pathway with individual genes at 4 h comparing CP (collagen peptides) and PLA (placebo). Color label indicates genes that are up (red) or down (green) regulated compared to PLA. Dark coloring = *p*-value equal or below 0.05, light coloring = *p*-value higher than 0.05.

Previous research shows that the phosphorylation levels of Akt and PI3K-Akt are significantly elevated following an acute bout of resistance exercise (Dreyer et al., 2008; Camera et al., 2010; Horii et al., 2020) and that this response can be augmented by protein supplementation (Dreyer et al., 2008; Camera et al., 2015; Lane et al., 2017). Besides being stimulated by whey protein (Lane et al., 2017), Akt protein has recently been shown to be upregulated following the ingestion of CP in combination with chronic resistance exercise training (12 weeks) (Oertzen-Hagemann et al., 2019). The results of the present study complement these previous findings and demonstrated that PI3K and Akt mRNA are significantly upregulated following an acute bout of high-load exercise when ingesting a CP supplement compared to placebo. After inspection of relevant genes which might be involved in PI3K-Akt pathway upregulation following CP, angiopoietin-1 seems to play key roles as important activators of the PI3k signaling (Figure 4). In previous animal models, McClung et al. (2015) demonstrated that muscle cell derived angiopoietin-1 is involved in promoting myoblast differentiation and myotube formation. These findings are supported by studies from Mofarrahi et al. (2015) confirming strong positive associations of angiopoietin-1 and muscle regeneration following fiber injury. Interestingly, previous findings point towards the notion that the PI3K-Akt pathway plays an essential role in the expression of type I collagen induced by TGF-beta (Yokoyama et al., 2012). Although speculative, it might be assumed that the improvement in extracellular connective tissue might help to facilitate an enhanced contractile force transmission from the muscle to tendons or bones (Holwerda and van Loon, 2021). Indeed, such improvements in muscle strength and force have repeatedly been demonstrated in previous experiments following prolonged CP intake in combination with resistance training (Zdzieblik et al., 2015; Jendricke et al., 2019).

**FIGURE 5:**
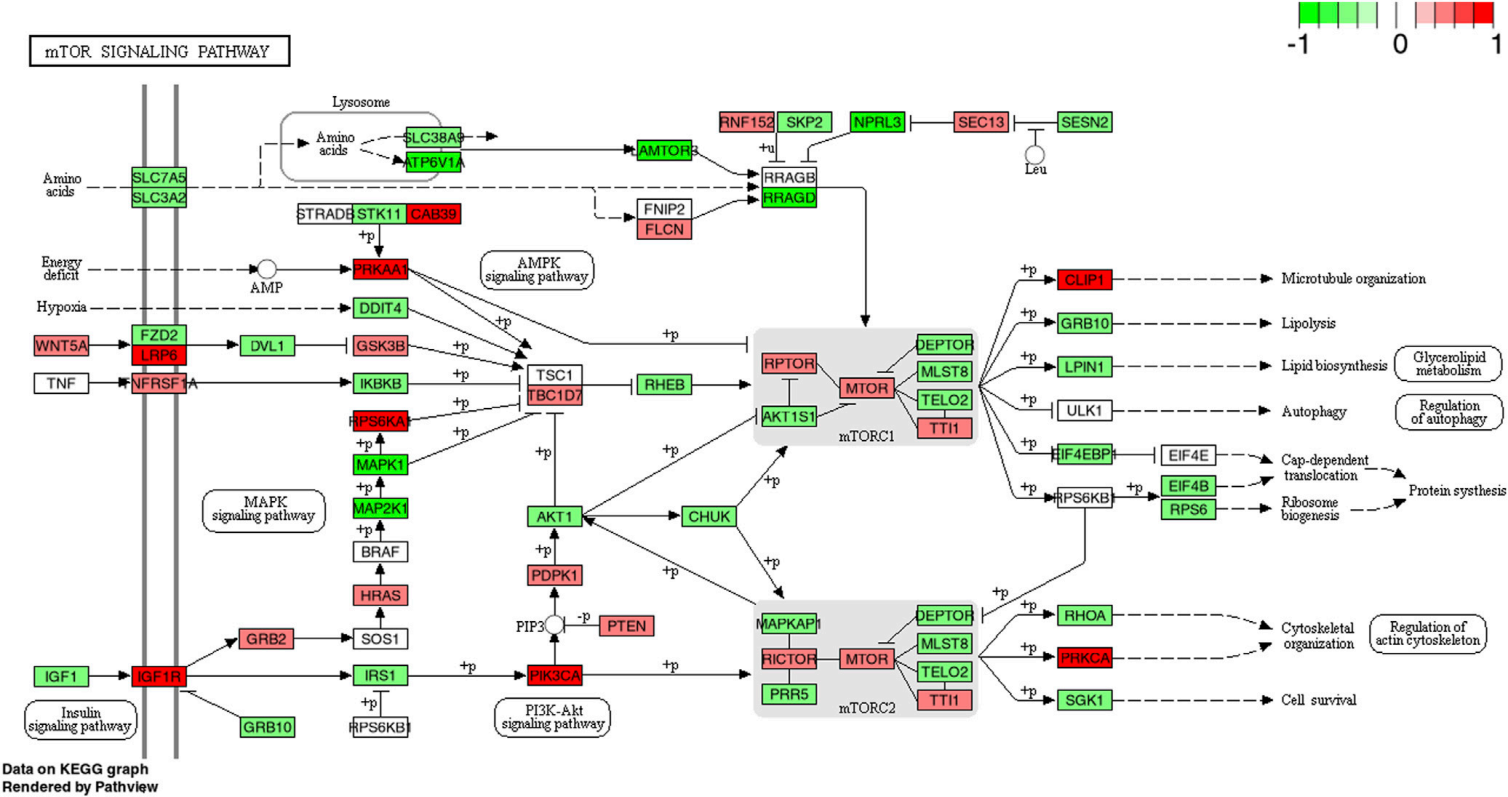
KEGG pathway of the mTOR signaling pathway with individual genes at 4 h comparing CP (collagen peptides) and PLA (placebo). Color label indicates genes that are up (red) or down (green) regulated compared to PLA. Dark coloring = *p*-value equal or below 0.05, light coloring = *p*-value higher than 0.05.

The mechanistic target of rapamycin (mTORC1) signaling pathway is generally considered as an important regulator of translation initiation (Bodine et al., 2001; Hoppeler, 2016). Besides the stimulatory effects of resistance exercise on the mTOR pathway (Drummond et al., 2009; Dreyer et al., 2010), numerous studies have also found favorable effects of protein supplementation manifested as an augmentation of mTOR phosphorylation and activation (Farnfield et al., 2009; Farnfield et al., 2012; Kakigi et al., 2014). Despite a visual increase, the present mRNA expression data demonstrated similar findings to these previous results, however, no statistical group by time effects were identified. A potential explanation for these results may lay within the temporal upregulation of the investigated pathways. Previous researchers confirmed that Akt acts as a precursor of mTOR by phosphorylating (and thus inhibiting) TSC2, a known suppressor of mTOR (Inoki et al., 2002). Given the present upregulation of PI3K-Akt gene expression at 4 h post exercise combined with CP intake, it may be speculated that mTOR upregulation peaked between 4 and 24 h and was thus missed due to the specific timing of sampling in the current study. Additionally, it could be speculated that the previously observed effects of longitudinal CP supplementation combined with resistance training on fat-free mass (Zdzieblik et al., 2015; Jendricke et al., 2019; Kirmse et al., 2019; Jendricke et al., 2020; Zdzieblik et al., 2021) can be explained *via* the stimulation of pathways other than the mTOR pathway (e.g., MAPK). The hypothesis of a potential convergence of mTOR and MAPK pathways to promote enhancements in anabolic signaling has been formulated by previous authors (Burd et al., 2010) although this aspect warrants further investigation. The exact mechanisms by which CP induce upregulations in gene expression remain largely unknown (Figure 5). Within the physiological chain of how upregulated gene expression pathways might facilitate physiologically relevant effects, Kitakaze et al. (2016) showed an increased upregulation of ribosomal S6 kinase which might facilitate increases in mRNA translation *via* eukaryotic initiation factor 3. Further studies are, however, needed to elucidate the physiological mechanisms in this field.

The MAPK-pathway has been shown to be involved in myofibrillar muscle protein synthesis as well as indirectly in collagen synthesis (Kimoto et al., 2004). A study by Holm et al. (2009) has shown that several steps in the MAPK pathway such as ERK1/2 or p38 are upregulated after exercise in an intensity-dependent manner and may therefore be reflective of the magnitude of mechanical stress applied to the muscle fiber (Martineau and Gardiner, 2001). Besides exercise-induced stimulation of the MAPK pathway (Williamson et al., 2003; Tannerstedt et al., 2009) there is conflicting evidence regarding the effects of protein feeding. In an early study by Karlsson et al. (2004) the authors found that the intake of branched-chained amino acids did not significantly augment the phosphorylation of several proteins found within the MAPK pathway (e.g., ERK1/2, p38) compared to resistance exercise alone. This is supported by a study from Moore and others (2011) indicating no effect of protein feeding alone but only when exercise was performed. Conversely, following a long-term trial with 12-weeks of resistance training and concomitant ingestion of CP, Oertzen-Hagemann et al. (2019) performed muscle proteome analyses and found that the MAPK pathway was overrepresented in the CP group compared to PLA. Although changes in muscle proteome and gene expression are not necessarily inter-related, these findings were confirmed by the present data, showing a significantly higher upregulation of MAPK mRNA in CP compared to PLA at 4 h post-exercise.

**FIGURE 6:**
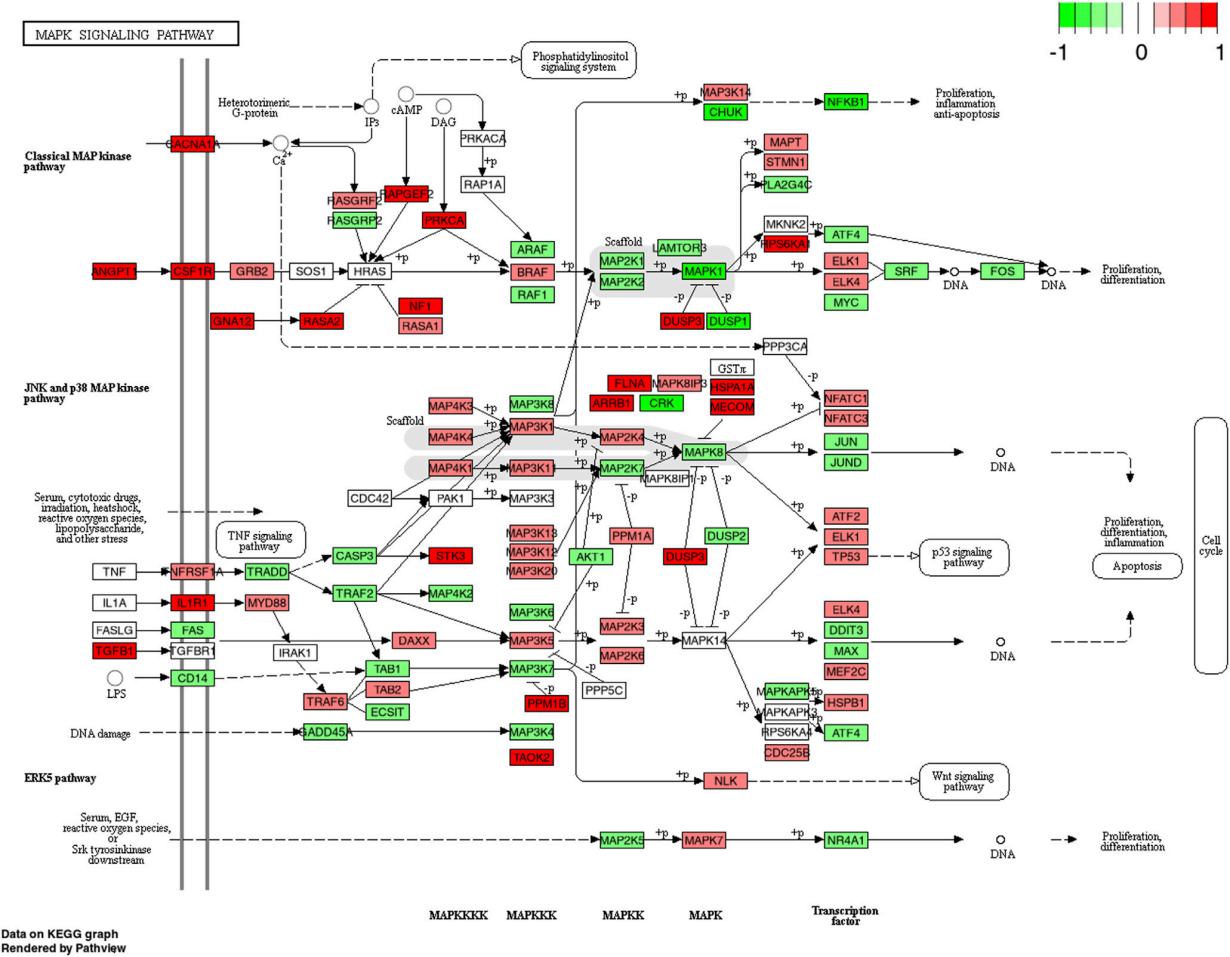
KEGG pathway of the MAPK signaling pathway with individual genes at 4 h comparing CP (collagen peptides) and PLA (placebo). Color label indicates genes that are up (red) or down (green) regulated compared to PLA. Dark coloring = *p*-value equal or below 0.05, light coloring = *p*-value higher than 0.05.

Interestingly, besides angiopoietin-1, upregulations of the MAPK pathway following CP ingestion seem to be driven by TGF-β-1 (Figure 6). TGF-β-1 is a multifunctional cytokine being involved in various cellular processes including type I collagen expression (Ismaeel et al., 2019) or myofibroblast transdifferentiation (Fan et al., 1999). In an earlier study by Heinemeier et al. (2003), the authors conclude that TGF-β-1 may play an important role in collagen formation after mechanical loading of tendon structures. As the PI3K-Akt and MAPK pathways are strongly inter-related, functional effects of extracellular matrix might facilitate optimized contractile force transmissions seen with long-term CP supplementation (Zdzieblik et al., 2015; Jendricke et al., 2019). This link, however, needs to be confirmed in future studies.

Although speculative, it may be argued that MAPK might be involved in mediating the effects on fat-free mass previously reported following 12 weeks of combined resistance training and CP supplementation (Zdzieblik et al., 2015; Jendricke et al., 2019; Jendricke et al., 2020; Zdzieblik et al., 2021). Such changes in fat-free mass might be explained by increases in either contractile or non-contractile elements. Generally, it must be mentioned that most studies focusing on intramuscular signaling following resistance exercise and protein supplementation investigated changes on the protein level. Therefore, further studies are needed which investigate the link between upregulated gene expression and myocellular as well as ECM protein synthesis. Conclusively, it needs to be mentioned that the findings from the present study are valid for this specific collagen peptide composition but might not necessarily be transferred to other collagen peptide ingredients.

## Perspectives

The main objective was to examine important gene expression pathways involved in muscular remodeling. The choice of these specific pathways was based on previous experiments (Oertzen-Hagemann et al., 2019) highlighting increased upregulations of proteins associated with protein metabolism and contractile fibers. It is important to note that the anabolic response to nutrition and exercise is highly dynamic and the observed acute changes are not necessarily predictive of long-term adaptations. Moreover, the current study investigated young and recreationally active males and the acute responses in healthy individuals cannot readily be transferred to responses in clinical cohorts. Nevertheless, this might also be of high clinical relevance for patients suffering from sarcopenia or diseases associated with muscular atrophy. To obtain a high level of standardization, individuals in this study were fasted (except CP and PLA intake) between the baseline and 4 h biopsy to exclude other mediating factors resulting from additional nutritional protein sources which might influence the results obtained. Conclusively, future studies are needed which validate these findings in further study populations including gender, age and training status as well as long-term effects including pre-exercise meals.

## Conclusion

The present study provides evidence for an increased upregulation of gene expression in the PI3K-Akt and MAPK pathways at 4 h following acute high-load resistance exercise combined with ingestion of CP compared to PLA. These results demonstrate for the first time that CP directly increase gene expression signaling of anabolic pathways in human skeletal muscle in response to an acute bout of resistance exercise. However, further studies, are needed to investigate the effects of combined resistance exercise and CP supplementation on PI3K-Akt and MAPK post-translational activation or miRNA regulation.

## Data Availability

The datasets presented in this study can be found in online repositories. The names of the repository/repositories and accession number(s) can be found below: https://www.ncbi.nlm.nih.gov/geo/, GSE195585.

## Abbreviations

1RM, One-repetition maximum; CP, Collagen peptides; ES, Enrichment score; FOXO, Forkhead-Box-Protein; GSEA, Gene set enrichment analysisl; MAPK, Mitogen-activated protein kinase; mTOR, Mechanistic target of rapamycin; MuRF1, Muscle RING-finger protein-1; NES, Normalized enrichment score; VL, Vastus lateralis muscle.
